# Supplementary material for: The Systems Biology Research Tool: evolvable open-source software
Source: BMC Syst Biol. 2008 Jun 29;2:55. doi: 10.1186/1752-0509-2-55 (PMC2446383; doi:10.1186/1752-0509-2-55)
Supplement: Additional file 1 — SBRT Archive. An archive of the current version of the Systems Biology Research Tool. [file 1752-0509-2-55-S1.zip › sbrt-1.4.0/doc/developers_guide/api/sbrt/shell/text/math/package-use.html]

Uses of Package sbrt.shell.text.math


|  |  |  |
| --- | --- | --- |
| **Overview** \| **Package** \| Class \| **Use** \| **Tree** \| **Deprecated** \| **Index** \| **Help** | |  |
| PREV   NEXT | **FRAMES**    **NO FRAMES**     **All Classes** |  |


---


## **Uses of Package sbrt.shell.text.math**

| Packages that use sbrt.shell.text.math | |
| --- | --- |
| **sbrt.shell.mng.graph\_theory** | Provides classes and for creating graph theoretical process managers. |
| **sbrt.shell.text.chem** | Provides classes and interfaces for defining formats for chemistry related objects. |
| **sbrt.shell.text.fba** | Provides classes and interfaces for defining formats for objects relevant to Flux Balance Analysis. |
| **sbrt.shell.text.math** | Provides classes and interfaces for defining formats for mathematical objects. |

Classes in sbrt.shell.text.math used by sbrt.shell.mng.graph\_theory

****DirEdgeFormat****
  
          This interface is used to represent formats for the edges of directed graphs.

Classes in sbrt.shell.text.math used by sbrt.shell.text.chem

****LinearCombFormatV1****
  
          This class is a concrete implementation of `LinearCombFormat`.

****LinearCombFormatV2****
  
          This class is a concrete implementation of `LinearCombFormat`.

Classes in sbrt.shell.text.math used by sbrt.shell.text.fba

****LinearCombFormatV1****
  
          This class is a concrete implementation of `LinearCombFormat`.

Classes in sbrt.shell.text.math used by sbrt.shell.text.math

****CoeffFormat****
  
          This interface is used to represent the format of
coefficients of mathematical expressions.

****CoeffFormatV1****
  
          This class is a concrete implementation of
`CoeffFormat`.

****CoeffFormatV2****
  
          This class is a concrete implementation of
`CoeffFormat`.

****CorrResultsFormatter****
  
          This interface is used to format the results of a correlation computation.

****DirEdgeFormat****
  
          This interface is used to represent formats for the edges of directed graphs.

****DirEdgeFormatV1****
  
          This class is a concrete implementation of `DirEdgeFormat`.

****IndexPairFormat****
  
          This interface is used to represent formats for pairs of indices.

****IndexPairFormatV1****
  
          This class is a concrete implementation of `IndexPairFormat`.

****IntervalFormat****
  
          This interface is used to represent formats for closed
intervals.

****IntervalFormatV1****
  
          This class is a concrete implementation of `IntervalFormat`.

****LinCombTermFormatV1****
  
          This class is a concrete implementation of `LinearCombTermFormat`.

****LinCombTermFormatV2****
  
          This class is a concrete implementation of `LinearCombTermFormat`.

****LinearCombFormat****
  
          This interface is used to represent formats for linear
combinations.

****LinearCombFormatV1****
  
          This class is a concrete implementation of `LinearCombFormat`.

****LinearCombFormatV2****
  
          This class is a concrete implementation of `LinearCombFormat`.

****LinearCombTermFormat****
  
          This interface is used to represent the formats of terms from
linear combinations.

****MathExprFormat****
  
          This interface is used to represent formats for linear
combinations.

****MatrixElementFormat****
  
          This interface is used to represent formats for matrix elements.

---


|  |  |  |
| --- | --- | --- |
| **Overview** \| **Package** \| Class \| **Use** \| **Tree** \| **Deprecated** \| **Index** \| **Help** | |  |
| PREV   NEXT | **FRAMES**    **NO FRAMES**     **All Classes** |  |


---
